# Complete Genome Sequence of *Marinobacterium* sp. Strain LSUCC0821, Isolated from the Coastal Gulf of Mexico

**DOI:** 10.1128/MRA.01035-20

**Published:** 2020-12-03

**Authors:** Anna M. Lucchesi, Michael W. Henson, Ben Temperton, J. Cameron Thrash

**Affiliations:** a Department of Biological Sciences, Louisiana State University, Baton Rouge, Louisiana, USA; b Department of Biological Sciences, University of Southern California, Los Angeles, California, USA; c School of Biosciences, University of Exeter, Exeter, United Kingdom; Georgia Institute of Technology

## Abstract

Here, we present the complete genome sequence of *Marinobacterium* sp. strain LSUCC0821, isolated from the coastal Gulf of Mexico with artificial seawater using high-throughput dilution-to-extinction (DTE) cultivation. The 2.36-Mbp circularized genome sequence has 2,231 predicted genes, a 91.5% coding density, and a GC content of 47.8%.

## ANNOUNCEMENT

*Marinobacterium* sp. strain LSUCC0821 was cultured from Bay de Pomme D’Or (Louisiana, Gulf of Mexico) in January 2017 using dilution-to-extinction (DTE) cultivation and artificial seawater (1, 2). The genus *Marinobacterium* (order *Oceanospirillales*) has been found in diverse environments, including oil seeps, tidal flats, and coral mucus (3–5). A BLASTn (default settings) search of the 16S rRNA gene sequence (1) against the NCBI nucleotide (nt) database showed the closest cultured representatives of LSUCC0821 (GenBank accession number MK603728) to be *Marinobacterium* sp. IMCC1424 (98% identity; KF146347) and *Marinobacterium marisflavum* IMCC4074 (97% identity; NR_125520) (6). We sequenced the genome of strain LSUCC0821 because of its geographic novelty and ability to enhance future comparative genomic studies of an important marine clade.

Cells for short-read sequencing were revived from four cryostocks of the Thrash lab culture collection in sterile polycarbonate flasks containing 50 ml of MWH3 medium (1). From one revival culture, two additional replicate cultures were grown in 500 ml of MWH3 medium to obtain enough biomass for long-read sequencing. All cultures were grown to peak cell density and filtered onto 0.2-μm polycarbonate filters (Pall, USA). DNA was extracted using lysozyme digestion, phenol-chloroform extraction, and isopropanol precipitation (7, 8). Prior to library preparation, DNA for short- and long-read sequencing was cleaned and concentrated using the genomic DNA clean and concentrator kit (Zymo Research, USA) following the manufacturer’s instructions. Short-read library preparation and sequencing were completed at the USC Genome Core (University of Southern California, USA). Briefly, the libraries were constructed using the KAPA HyperPlus library preparation kit (Kapa Biosystems, Inc., USA), quantified using the Qubit fluorometer, and analyzed for size and quality using the Agilent BioAnalyzer system. The library DNA was pooled equimolarly and sequenced on an Illumina NextSeq 550 instrument, using a midoutput flow cell in paired-end (PE) 150-bp cycle format, generating 13,776,782 reads. The long-read sequencing libraries were prepared using the SQK-LSK108 1D genomic DNA ligation kit (Oxford Nanopore, UK) with slight modifications (available at https://doi.org/10.17504/protocols.io.bixskfne) and sequenced on a MinION instrument using an R9.4 flow cell (Oxford Nanopore), generating 247,039 raw reads with an *N*_50_ value of 7,955 bp. FAST5 sequences were base called with Guppy v.2.3.1 (Oxford Nanopore Technologies) using flipflop mode. Adapters and split reads with adapters in the middle were removed using Porechop (https://github.com/rrwick/Porechop) with the flags “require_two_barcodes” and “discard_middle.”

Genome assembly was completed using long and short reads with Unicycler v.0.4.8-beta with default settings (9), resulting in a single circular scaffold (rotation and circularity confirmed via Unicycler; assembly graph available at https://doi.org/10.6084/m9.figshare.12857879). The contamination and quality were assessed using CheckM v.1.0.3 with default settings (10). The assembled genome sequence was annotated using the NCBI Prokaryotic Genome Annotation Pipeline (11). The completed genome sequence of LSUCC0821 is 2,360,824 bp (105× coverage), with a GC content of 47.8%, a coding density of 91.5%, and estimated contamination of 0.43%. There are 2,303 predicted genes, 54 tRNA genes, and 3 each of the 5S, 16S, and 23S rRNA genes.

### Data availability.

Sample information, fastq sequences, and genomic assembly/annotation are accessible under the NCBI BioProject and whole-genome sequence (WGS) accession numbers PRJNA589095 and CP051666, respectively. Cryostocks and/or live cultures of LSUCC0821 are available upon request.

## References

[1] Henson MW, Lanclos VC, Pitre DM, Weckhorst JL, Lucchesi AM, Cheng C, Temperton B, Thrash JC 2020 Expanding the diversity of bacterioplankton isolates and modeling isolation efficacy with large scale dilution-to-extinction cultivation. Appl Environ Microbiol 86:e00943-20. doi:10.1128/AEM.00943-20. 32561583 PMC7440811

[2] Henson MW, Pitre DM, Weckhorst JL, Lanclos VC, Webber AT, Thrash JC 2016 Artificial seawater media facilitate cultivating members of the microbial majority from the Gulf of Mexico. mSphere 1:e00028-16. doi:10.1128/mSphere.00028-16. PMC4894692 27303734

[3] Rosano-Hernández MC, Fernández-Linares LC, Xoconostle-Cazares B 2009 Bacterial diversity of marine seeps in the southeastern Gulf of Mexico. Pak J Biol Sci 12:683–689. doi:10.3923/pjbs.2009.683.689. 19634471

[4] Park S, Jung Y-T, Kim S, Yoon J-H 2016 Marinobacterium aestuariivivens sp. nov., isolated from a tidal flat. Int J Syst Evol Microbiol 66:1718–1723. doi:10.1099/ijsem.0.000927. 26812956

[5] Chimetto LA, Cleenwerck I, Brocchi M, Willems A, De Vos P, Thompson FL 2011 Marinobacterium coralli sp. nov., isolated from mucus of coral (Mussismilia hispida). Int J Syst Evol Microbiol 61:60–64. doi:10.1099/ijs.0.021105-0. 20154332

[6] Kim H, Oh H-M, Yang S-J, Lee J-S, Hong J-S, Cho J-C 2009 Marinobacterium marisflavi sp. nov., isolated from a costal seawater. Curr Microbiol 58:511–515. doi:10.1007/s00284-009-9355-5. 19189183

[7] Raymond JA, Christner BC, Schuster SC 2008 A bacterial ice-binding protein from the Vostok ice core. Extremophiles 12:713–717. doi:10.1007/s00792-008-0178-2. 18622572

[8] Murray MG, Thompson WF 1980 Rapid isolation of high molecular weight plant DNA. Nucleic Acids Res 8:4321–4325. doi:10.1093/nar/8.19.4321. 7433111 PMC324241

[9] Wick RR, Judd LM, Gorrie CL, Holt KE 2017 Unicycler: resolving bacterial genome assemblies from short and long sequencing reads. PLoS Comput Biol 13:e1005595. doi:10.1371/journal.pcbi.1005595. 28594827 PMC5481147

[10] Parks DH, Imelfort M, Skennerton CT, Hugenholtz P, Tyson GW 2015 CheckM: assessing the quality of microbial genomes recovered from isolates, single cells, and metagenomes. Genome Res 25:1043–1055. doi:10.1101/gr.186072.114. 25977477 PMC4484387

[11] Tatusova T, DiCuccio M, Badretdin A, Chetvernin V, Nawrocki EP, Zaslavsky L, Lomsadze A, Pruitt KD, Borodovsky M, Ostell J 2016 NCBI Prokaryotic Genome Annotation Pipeline. Nucleic Acids Res 44:6614–6624. doi:10.1093/nar/gkw569. 27342282 PMC5001611

